# Carbolic Acid

**Published:** 1880-07

**Authors:** J. G. Westmoreland

**Affiliations:** Atlanta, Ga.


ATLANTA
Medical and Surgical Journal.

VOL. XVIII.] JULY—1880. [No. 4.

### Original Communications.

### CARBOLIC ACID.

BY J. G. WESTMORELAND, M.D., ATLANTA, GA.

From time to time, for several years, we have, through this Journal, and otherwise called attention to the important actions of this remedy. To its local action I now desire again, particularly, to refer. Reputed cancer curers are generally the most detestible of itenerant quacks, and hasten the destruction of parts affected with cancerous ulceration without at all arresting or changing the unnatural condition. The remedies usually applied destroy any kind of tissue, whether contaminated or not, and add to, rather than arrest the destructive action going on. The result is that growths and ulcers exhibiting the true cancer cell are looked upon as incurable. Even where the part affected can be entirely removed by the knife or other means, the prevailing opinion is that the cancerous diathesis still existing leaves the subject liable to a similar condition in some other part.

These facts lead scientific physicians to believe cancer incurable, and hence the progeny even of those affected with it are considered unsafe from this fatal disease.

It is not my intention to deny the facts or attempt to refute these theories. I desire only to call attention again to the action of carbolic acid in this and other forms of ulceration and inflammation.

Nitric, chromic and sulphuric acids destroy all soft tissues with which they come in contact, whether unsound or healthy, and do so at once. Arsenic acid, more slowly, but with equal certainty, does the same thing. Not so with carbolic acid. Destructive action is very slightly, if at all had upon sound tissues, by the application of a moderate quantity. The mucous membranes particularly resist corrosion well. Even the mouth of an infant receives no injurious impression from contact with the undiluted acid. While contraction of parts from corrosive acids is an ordinary result, leading to stenosis and stricture of canals, no such trouble need be apprehended from carbolic acid. Having tested it thoroughly, by the introduction into the urethra, the vagina and whole interior of the uterus, the writer can testify as to its perfect safety when used in this way. Not only is there no contraction from cicatricial tissue, but the pain produced by it is not so severe as by the ordinary strength of solution nitrate of silver, iodine, zinc, &c , used for chronic inflammation of these parts. Strange to say, however, the pudendum, glands penis and prepuce are painfully affected by the escape of acid from the canals upon these external parts.

The object of this article is not so much to call attention to the use of the acid in the treatment of urethral, vaginal and uterine chronic inflammation, in which I have been in the habit of using it in the strength of from 60 grains to the ounce, to full strength, but the very favorable effects obtained from it in the treatment of chirrous malignant ulcers.

The first case to which I advised the application was one of cauliflower ulcer of the os uteri, but owing, as I now think, to the imperfect manner of its use, no favorable report was made, and the patient finally died of the disease. Having advised it in this case without a precedent, and receiving no encouragement from the result, I was not hasty in seeking another subject of the kind for its use. About one year ago, however, in consultation with Dr. E. Griffin, of this county, a case was presented of similar character. The usual remedies for non-malignant ulcer, such as iodine, nitrate of silver, with weak solution carbolic acid, failing to arrest the ulceration and hemorrhage, the doctor, in my absence, made application of undiluted acid with very satisfactory effect. It was continued at intervals of four or five days, with rapid improvement. In a month from the first application the ulcer had entirely healed, and the lady, who had been confined to her room, was able to visit, attend her household affairs, etc., and has not, up to this time, had any return of the disease.

About the first of January last I was called to see Mrs. F., of this city, not with any hope of cure, but for the purpose of giving temporary relief from pain, sooth the sufferer and smooth her passage to the grave. The history showed that for several months she had been confined to her room, and that hemorrhage was almost constant with occasional attacks of alarming discharge. The patient was confined strictly to the bed, and gave evidences of anæmia and decided prostration. Digital examination revealed an indurated and rough condition of the whole vagina and os and neck of the womb, with hemorrhagic tendency throughout.

The speculum showed a bleeding, rough ulcer from the os externum to, and including the os and cervis uteri. In addition, diarrhæal discharges with tendency to dysentery were frequent; appetite poor and digestion bad.

The treatment was commenced with opium, blue mass and tannin, to correct the bowel trouble, and the application of carbolic acid in full strength to the ulcerated surface. The crystals were reduced to the liquid form by heat, and saturating a pledget of soft old linen, they were applied to the ulcer along its whole extent, using a fresh piece, as one would become exhausted of the acid. This was repeated every fourth, and sometimes every third day, requiring not less than half a fluid ounce for each application. The effect not only exceeded all expectation, but proved, indeed, marvelous. In three months nearly half tne ulcerated surface had entirely healed, with the formation of perfect mucous membrane, with very marked relief to the induration throughout, and entire cessation of hemorrhage. The patient's general health and strength had so much improved that she was able to be up most of the day and walk about the house and yard with ease and comfort.

I was frequently interrogated by the patient and her friends as to the nature of the disease and probability of perfect recovery; and while I freely expressed my surprise and pleasure at her decided and continued improvement, I could not, with all previous experiences in such cases before me, promise permanent recovery. At this juncture of affairs the fair promises of others to make a perfect cure, and among them a female cancer curer, enticed her to discontinue my treatment.

To-day I called to see her, and found her, in appearance, about the same as when I left her three months ago. I made no examination of the diseased organ, but was informed that some hemorrhage has occurred two or three times, and that the occasional difficulty in urinating which existed during my treatment from induration about the urethra, is still sometimes present. The digestion is poor, and occasional attacks of diarrhœa occur, as when she was under my care. From these facts I infer that the disease has made very little advancement in my absence, but is perhaps slowly returning to its original condition, as I anticipated when leaving.

The result of treatment in this case gives me confidence in the use of carbolic acid in recent cancerous ulceration of the os uteri and elsewhere. The remedy being a first rate styptic and antiseptic, its use will well compensate for any trouble in the application, for the prevention of hemorrhage and unpleasant odor, if no permanent relief from the disease be affected. Moreover, the acute pain which is so distressing in cancerous ulcers, is, in a great measure, relieved by the acid.

Such cases being frequently met with, ample opportunity is afforded those who would like to test the efficacy of this remedy; and if it should be found only useful as it proved in this imperfect test, we shall have made decided advancement in the management of this very disagreeable, painful and fatal disease.

The ulcerated surface, of whatever nature the ulcer may be, becomes perfectly whitened by the application, and it is proper, in order to protect the parts not ulcerated from unnecessrry irritation and soreness, that the free acid should be absorbed by sponge or soft cloth, and removed after every part of the disease has been whitened.

I will take great pleasure in recording in the JOURNAL the result of treatment in this way by any one who will make the test and send me the report.

